# *Mycobacterium lepromatosis* as a Second Agent of Hansen’s Disease

**DOI:** 10.3389/fmicb.2021.698588

**Published:** 2021-09-10

**Authors:** Patrícia Deps, Simon M. Collin

**Affiliations:** ^1^Department of Social Medicine, Universidade Federal do Espírito Santo, Vitória, Brazil; ^2^Postgraduate Programme in Infectious Diseases, Universidade Federal do Espírito Santo, Vitória, Brazil; ^3^National Infection Service, Public Health England, London, United Kingdom

**Keywords:** *Mycobacterium lepromatosis*, *Mycobacterium leprae*, Hansen’s disease, zoonosis, leprosy

## Abstract

*Mycobacterium lepromatosis* was identified as a new species and second causal agent of Hansen’s disease (HD, or leprosy) in 2008, 150years after the disease was first attributed to *Mycobacterium leprae*. *M. lepromatosis* has been implicated in a small number of HD cases, and clinical aspects of HD caused by *M. lepromatosis* are poorly characterized. HD is a recognized zoonosis through transmission of *M. leprae* from armadillos, but the role of *M. lepromatosis* as a zoonotic agent of HD is unknown. *M. lepromatosis* was initially associated with diffuse lepromatous leprosy, but subsequent case reports and surveys have linked it to other forms of HD. HD caused by *M. lepromatosis* has been reported from three endemic countries: Brazil, Myanmar, and Philippines, and three non-endemic countries: Mexico, Malaysia, and United States. Contact with armadillos in Mexico was mentioned in 2/21 *M. lepromatosis* HD case reports since 2008. *M. lepromatosis* in animals has been investigated only in non-endemic countries, in squirrels and chipmunks in Europe, white-throated woodrats in Mexico, and armadillos in the United States. To date, there have only been a small number of positive findings in Eurasian red squirrels in Britain and Ireland. A single study of environmental samples found no *M. lepromatosis* in soil from a Scottish red squirrel habitat. Future studies must focus on endemic countries to determine the true proportion of HD cases caused by *M. lepromatosis*, and whether viable *M. lepromatosis* occurs in non-human sources.

## Introduction

Hansen’s disease (HD) or leprosy has been attributed to *Mycobacterium leprae* (*M. leprae*) since the late nineteenth century, after the discoveries of Gerhard Armauer Hansen. In 2008, bacilli from two HD cases were identified as a new species, *Mycobacterium lepromatosis* (*M. lepromatosis*; Han et al., 2008). These first two cases manifested as a specific multibacillary form of HD, diffuse lepromatous leprosy (DLL), co-occurring with Lucio’s phenomenon (LP), a severe HD reaction. Subsequent case reports indicated that *M. lepromatosis* was not related specifically to DLL, but also with other multibacillary forms of HD. Dual infection with *M. lepromatosis* and *M. leprae* has also been reported in case reports from South America (Aldama Olmedo et al., 2020) and South East Asia (Han et al., 2012a; Widiatma and Sukanto, 2019) and in surveys of specimens from Mexico and Brazil (Han et al., 2012b, 2014; Kai et al., 2016; Torres-Guerrero et al., 2018; Sharma et al., 2020). Medical and scientific literature refers to both species as the causal agents of HD, but cases attributed to *M. lepromatosis* infection are infrequently reported, and the clinical aspects of HD caused by *M. lepromatosis* remain poorly characterized.

In addition to this apparent commonality of *M. lepromatosis* and *M. leprae* in causing a single disease in humans, both mycobacterium species have been detected in mammals presenting with and without clinical signs of disease. For *M. leprae*, experimental and natural infection in armadillos is well-described (Oliveira et al., 2019). Indeed, armadillo species have served as a model for HD in humans since the 1970s (Storrs, 1971; Sharma et al., 2013). A meta-analysis of *M. leprae* infection in wild armadillos in Brazil found a pooled prevalence from 8 PCR-based studies conducted in seven states equivalent to one in ten animals being infected, with three studies reporting no infection and one study in a hyperendemic area of a northeastern state reporting infection in 20/20 armadillos (Deps et al., 2020). Apart from armadillos, natural *M. leprae* infection in wild (non-captive) animals has been reported only in red squirrels from the British Isles and Ireland (Ploemacher et al., 2020), and more recently, in two chimpanzees in West Africa (Hockings et al., 2020).

From a One Health perspective, HD in the United States is recognized as a zoonosis (Centers for Disease Control and Prevention, 2020), with genomic evidence linking *M. leprae* from armadillos in south eastern states with sporadic cases of HD (Truman et al., 2011; Sharma et al., 2015). In Brazil, where HD is endemic and hunting and consumption of armadillos is widespread, zoonotic transmission is generally regarded to be of little or no concern given the presumed predominance of human-to-human transmission, despite evidence that exposure to armadillos confers additional HD risk even in highly endemic areas (Deps et al., 2021). *M. leprae* has also been detected in environmental samples (Ploemacher et al., 2020), including in soil and water in areas endemic for HD in India (Mohanty et al., 2016; Singh et al., 2020), and from water sources in endemic areas in Brazil (Holanda et al., 2017).

In this article, we review the emergence of *M. lepromatosis* as a cause of HD in humans and evidence to date for the existence of *M. lepromatosis* in animal hosts and environmental reservoirs, and we discuss, in the context of evidence for *M. leprae* as a zoonotic pathogen, what steps need to be taken to determine the prevalence of *M. lepromatosis* infection in human and animals and whether *M. lepromatosis* might be a zoonotic source of HD.

## *Mycobacterium lepromatosis* as a Cause of Disease in Humans

### Discovery and First Cases

The new mycobacterium species *M. lepromatosis* was identified and named by Han et al. (2008) in 2008 following the death of a patient of Mexican origin who had been diagnosed with DLL and LP (Han et al., 2008). Sequencing by PCR of the ~1500bp 16S ribosomal RNA (rRNA) gene in acid-fast bacilli (AFB) from frozen liver autopsy specimens showed that the AFB strain (designated FJ924) had a closest match with *M. leprae* (1475/1506bp, 97.9%) and a second closest match with *M. haemophilum* (1465/1505, 97.3%). Strain FJ924 had a 19bp AT-rich inserted sequence in its 16S rRNA gene which was not found in any other bacterial species, just as *M. leprae* has a unique short (16bp) AT-rich sequence inserted in its 16S rRNA gene. The researchers obtained archived biopsy specimens from a second patient of Mexican origin who had died 5years previously, also with DLL and LP. Gene sequences from this earlier case, including of the 16S rRNA gene, matched 100% with strain FJ924.

On the basis of these results, Han et al. (2012a) proposed a new species, *M. lepromatosis*, as a causal agent of DLL, while speculating that it might also cause lepromatous (LL) and borderline lepromatous (BL) forms of HD (Han et al., 2008). At the time of their first study, the researchers obtained archived specimens from two fatal cases of DLL in Singapore (both patients died in 1999). PCR using primers for the unique 16S rRNA inserted sequences, LPMF2 for *M. lepromatosis* and LERF2 for *M. leprae*, was positive for both species in both patients, indicating dual infection (Han et al., 2012a).

### Subsequent Case Reports and Specimen Surveys

Case reports since 2008 have identified *M. lepromatosis* infection in a further 15 patients (Vera-Cabrera et al., 2011; Jessamine et al., 2012; Han and Jessurun, 2013; Han and Quintanilla, 2015; Sotiriou et al., 2016; Velarde-Felix et al., 2016; Cleary et al., 2017; Virk et al., 2017; Htet et al., 2018; Flores-Suarez et al., 2019; Trave et al., 2020; Watson et al., 2020), plus two patients with dual infections of *M. lepromatosis* and *M. leprae* (Widiatma and Sukanto, 2019; Aldama Olmedo et al., 2020). Of the 21 *M. lepromatosis* cases, 11 (52.4%) patients were from Mexico (seven were United States residents), 2 each from Singapore, Myanmar, and United States, and one each from Indonesia, Paraguay, Cuba, and Canada. Five of the 21 case investigations used DNA from archived biopsy specimens, the earliest of which was a patient from Mexico who was diagnosed with DLL in 1963 and treated at Carville, United States (Han and Jessurun, 2013). Where reported, clinical presentation was DLL in 10 cases including six with LP, four were LL, including two with erythema nodosum leprosum (ENL), and two were BL.

In addition to case reports, 10 retrospective specimen surveys have examined 1,260 archived biopsy specimens, detecting *M. lepromatosis, M. leprae* or dual infection in, respectively, 106 (15.8%), 798 (84.6%), and 28 (3.0%) of 943 PCR-positive specimens (Han et al., 2012b, 2014; Singh et al., 2015; Yuan et al., 2015; Zhang et al., 2015; Kai et al., 2016; Torres-Guerrero et al., 2018; Bezalel et al., 2019; Masood et al., 2019; Sharma et al., 2020). *M. lepromatosis* was detected in 42.2% (116/275) of PCR-positive specimens from Mexico, in 12.7% (10/79) from Brazil, 4.8% (5/105) from south East Asia (Malaysia, Myanmar, and Philippines), and 1.5% (3/195) from the United States (Table 1). All of 157 specimens from China (Yuan et al., 2015; Zhang et al., 2015), 48 from Mali, and 77 from Venezuela were positive only for *M. leprae* (Singh et al., 2015).

**Table 1:** Survey findings of *Mycobacterium leprae* and *Mycobacterium lepromatosis* in Hansen’s disease and animal and environmental reservoirs.

	Country	*M. lepromatosis*	*M. leprae*	Reference
Human (Hansen’s disease)				
	Brazil^†^	12.7% (10/79)	87.3% (69/79)	Han et al., 2014; Singh et al., 2015
	Mexico^†^	42.2% (116/275)	53.8% (148/275)	Han et al., 2012b; Han et al., 2014; Singh et al., 2015; Vera-Cabrera et al., 2015; Kai et al., 2016; Torres-Guerrero et al., 2018; Sharma et al., 2020
	Venezuela	0.0% (0/77)	100.0% (77/77)	Singh et al., 2015
	United States	1.5% (3/195)	98.5% (192/195)	Masood et al., 2019; Sharma et al., 2020
	Mali	0.0% (0/48)	100.0% (48/48)	Singh et al., 2015
	South East Asia (Myanmar, Malaysia, and Philippines)	4.8% (5/105)	95.2% (100/105)	Han et al., 2014; Sharma et al., 2020
	China	0.0% (0/157)	100.0% (157/157)	Yuan et al., 2015; Zhang et al., 2015
Animal				
Eurasian red squirrel (*Sciurus vulgaris*)	England (Isle of Wight)	100.0% (1/1)	Not reported	Simpson et al., 2015
	England (Isle of Wight)	100.0% (1/1)	0.0% (0/1)	Avanzi et al., 2016
	England (Isle of Wight)	1.1% (1/92)	0.0% (0/92)	Butler et al., 2017
	England (Brownsea Island)	0.0% (0/25)	100.0% (25/25)	Avanzi et al., 2016
	Scotland (Isle of Arran)	13.6% (6/44)	0.0% (0/44)	Avanzi et al., 2016
	Ireland	5.0% (2/40)	0.0% (0/20)	Avanzi et al., 2016
	Netherlands	0.0% (0/61)	0.0% (0/61)	Tio-Coma et al., 2020
	Belgium	0.0% (0/53)	0.0% (0/53)	Tio-Coma et al., 2020
	Italy, Germany, France, and Switzerland	0.0% (0/96)	0.0% (0/96)	Schilling et al., 2019
Eastern gray squirrel (*Sciurus carolinensis*)	Scotland (Isle of Arran)	0.0% (0/4)	0.0% (0/4)	Avanzi et al., 2016
	Italy and UK	0.0% (0/67)	0.0% (0/67)	Schilling et al., 2019
Pallas’s squirrel (*Callosciurus erythraeus*)	Italy and France	0.0% (0/103)	0.0% (0/103)	Schilling et al., 2019
Siberian chipmunks (*Tamias sibiricus*)	France	0.0% (0/35)	0.0% (0/35)	Schilling et al., 2019
White-throated woodrats (*Neotoma albigula*)	Mexico	0.0% (0/72)	0.0% (0/72)	Schilling et al., 2019
Armadillo (*Dasypus novemcinctus*)	United States	0.0% (0/106)	100% (106/106)	Sharma et al., 2020
Environment				
Soil	England (Brownsea Island)	Not tested	10.0% (1/10)	Tió-Coma et al., 2019
	Scotland (Isle of Arran)	0.0% (0/10)	not tested	Tió-Coma et al., 2019
	India	Not tested	32.3% (191/592)	Lavania et al., 2008; Turankar et al., 2012, 2016; Mohanty et al., 2016; Singh et al., 2020
	Bangladesh	Not tested	16% (4/21)	Tió-Coma et al., 2019
	Suriname	Not tested	10.7% (3/25)	Tió-Coma et al., 2019
Water	India	Not tested	24.2% (41/169)	Mohanty et al., 2016
	Brazil	Not tested	76.7% (23/30)	Holanda et al., 2017

† Dual infection was reported in 3/79 specimens from Brazil and 25/275 specimens from Mexico.

Case report and specimen survey studies have demonstrated that *M. lepromatosis* can be found in patients with different forms of HD, including DLL, LL, and BL, and with leprosy reactions, including ENL and LP. Biases in case reporting and archived specimen selection, particularly from regions, such as Mexico where DLL appears to be more common, compounded by possible case misclassification (misdiagnosis), mean that currently available data cannot be used to test the hypothesis that *M. lepromatosis* is disproportionately associated with DLL and/or LP.

A potentially important methodological limitation of some of these case studies and specimen surveys that warrants an in-depth systematic review with detailed quality assessment is that *M. lepromatosis* was detected using a range of *M. lepromatosis* specific primers without confirmation by sequencing.

### Genomics and Phylogenetics

Phylogenetic analyses and genome sequencing of the original FJ924 strain (Han et al., 2009, 2015) [NCBI NZ_LAWX01000000] and of two strains of *M. lepromatosis* from HD cases in Mexico Mx1-22A (GenBank JRPY00000000.1) (Singh et al., 2015; and NHDP-385 (NCBI SAMN12872980) (Sharma et al., 2020), has determined that the *M. leprae* and *M. lepromatosis* genomes have near-perfect synteny and their protein-coding genes share 93% nucleotide sequence identity. Phylogenetic analysis indicates divergence from a most recent common ancestor approximately 10–14 millionyears ago (Han et al., 2009; Singh et al., 2015). *M. lepromatosis* is closest to *M. leprae* SNP type 3K strains, which represent the most ancestral lineage of *M. leprae* (Singh et al., 2015). Pseudogenes were ~80% identical between *M. leprae* and *M. lepromatosis*, and 84 genomic regions (>500 nucleotides) of *M. lepromatosis*, representing ~5% of the genome (~166kb) comprising mainly pseudogenes, have no counterparts in *M. leprae* (Singh et al., 2015). *M. lepromatosis* and *M. leprae* share the same repeat families (RLEP, REPLEP, LEPREP, and LEPRPT) at the same genomic locations, but their sequences have diverged substantially ranging from 75 to 90% sequence identity and proportional to copy number (Singh et al., 2015).

The few functional gene differences that have been identified to date do not provide evidence to support differences in the pathogenic properties of the two species, such as a putative greater propensity of *M. lepromatosis* to invade the cutaneous vascular endothelium (Singh et al., 2015). *M. leprae* genes encoding laminin-2 binding protein ML1683c and the six enzymes (ML0128, ML2348 ML0126, ML0127, ML23246c, and ML2347) required to produce the terminal trisaccharide moiety of phenolic glycolipid 1 (PGL-1) are highly conserved in *M. lepromatosis* (Singh et al., 2015), suggesting likely anti-PGL-1 seropositivity in *M. lepromatosis* HD cases and nerve involvement and damage through invasion of Schwann cells (Ng et al., 2000). One human and one squirrel case of *M. lepromatosis* infection were reported to be anti-PGL-1 seropositive (Avanzi et al., 2016). The *folP1*, *rpoB*, and *gyrA* drug resistance determining regions have high homology, but with sufficient mutations to require that drug sensitivity techniques developed for *M. leprae* are validated against *M. lepromatosis* (Kai et al., 2016; Araujo et al., 2017).

Notable genetic differences between the species included the presence in *M. lepromatosis* of the coproporphyrinogen III oxidase (*hemN*) gene, which is present in *M. tuberculosis* but absent from *M. leprae*, and relatively large variation in ESX-1 secreted protein genes associated with mycobacterial virulence (Ates et al., 2016). One of these, *espA* with 78% protein identity (Singh et al., 2015), codes for part of the LID-1 fusion protein developed as a serological test of HD (Duthie et al., 2020), raising the possibility that this test might be less sensitive for *M. lepromatosis* infection. Given concerns that the 98% identity of 16S rDNA sequences in the two species could yield unreliable PCR results when using primers for this region, the recent development and validation of a unique repetitive element PCR assay for *M. lepromatosis* (RLPM, equivalent to RLEP *M. leprae*) provide a reliable diagnostic method with which to investigate further this new species as a causative agent of HD (Sharma et al., 2020). RLPM is an approximately 200bp region of which there are 5–6 copies yielding an approximate limit of detection (LOD) of 3.0 *M. lepromatosis* bacilli per reaction (compared with LOD ≈0.8 for RLEP ~130bp sequence of which there are 29–36 copies in *M. leprae*; Sharma et al., 2020). The RLPM assay was demonstrated to be positive for *M. lepromatosis* but negative against 17 other mycobacterial species, including *M. leprae* and 10 other mycobacteria associated with human diseases (Sharma et al., 2020).

The RLPM and RLEP assays have been validated according to the Clinical Laboratory Improvement Amendments guidelines (Sharma et al., 2020), and their use should address the limitations described above regarding detection of *M. lepromatosis* in earlier studies.

## *Mycobacterium lepromatosis* as a Zoonotic Agent

### Animal Hosts

In animals, *M. lepromatosis* was first detected in Eurasian red squirrels (*Sciurus vulgaris*) from England, Ireland, and Scotland (Avanzi et al., 2016) (NCBI SRR3672737-SRR3672758, SRR3674396-SRR3674450, SRR3674451-SRR3674453, SRR3673933). These findings have not been repeated in Eurasian red, Eastern gray (*Sciurus carolinensis*), or Pallas’s (*Callosciurus erythraeus*) squirrels or in Siberian chipmunks (*Tamias sibiricus*) from other parts of Europe (Schilling et al., 2019; Tio-Coma et al., 2020). The prevalence of *M. lepromatosis* in the Scottish squirrel population 12.5% (6/48 squirrels) was lower than the 100% prevalence (25/25) of *M. leprae* in Brownsea Island squirrels (Tió-Coma et al., 2019; Table 1).

Phylogenetic analyses determined that the strain of *M. leprae* in squirrels in the British Isles was closest to strains that circulated in Medieval England (and which belong to the sequence type 3I branch of *M. leprae* found in wild armadillos in the United States), whereas the *M. lepromatosis* strain had diverged around 27,000years ago from a common ancestor of the strain recently identified in HD cases in Mexico (Avanzi et al., 2016).

In the United States, lymph node and spleen specimens from 106 wild 9-banded armadillos (*Dasypus novemcinctus*) found to be seropositive for *M. leprae* PGL-1 or LID-1 antigens in a previous study (Sharma et al., 2015) were all PCR positive for *M. leprae* and negative for *M. lepromatosis* (Sharma et al., 2020). Schilling et al. (2019) tested 72 Mexican white-throated woodrats (*Neotoma albigula*) obtained from a meat market in Monterrey, Mexico, for the presence of *M. leprae* and *M. lepromatosis*, and all PCR results were negative (Schilling et al., 2019). No studies to date have investigated wild animals in HD endemic countries (Table 1).

In summary, while evidence for armadillos as natural hosts of *M. leprae* is irrefutable, and natural *M. leprae* infection has also been detected in small numbers of red squirrels, the latter (from a single site in England) remain the only known natural hosts of *M. lepromatosis*.

### Environmental Reservoirs

In the British Isles, *M. leprae*-specific DNA was found in 1/10 soil samples from Brownsea Island, an area where *M. leprae* infection in red squirrels had previously been identified (Avanzi et al., 2016), but *M. lepromatosis* DNA could not be detected in soil samples from the Isle of Arran, where squirrels had been infected by *M. lepromatosis* (Avanzi et al., 2016; Tió-Coma et al., 2019). All other studies of water and soil have used methods only for detecting *M. leprae* (Table 1), providing conclusive evidence for persistence of *M. leprae* in the environment that is currently lacking for *M. lepromatosis*.

### Zoonotic Transmission

Among the case reports of HD caused by *M. lepromatosis*, zoonotic sources were suggested in two patients from Guerrero state, Mexico, who were diagnosed several years after emigrating to the United States, both with multibacillary forms of HD (one DLL and one BL). Both patients reported direct contact with armadillos (hunting, handling, and eating) when they lived in Mexico (Sotiriou et al., 2016; Cleary et al., 2017).

Outside the United States, history of contact with armadillos is not often asked of persons presenting with HD. However, in a survey of biopsy specimens from 38 HD patients currently under treatment in Nuevo Léon, Mexico, of 5 patients positive for *M. lepromatosis*, one DLL case reported eating armadillo and field rat (*rattus rattus*) meat and one BL case reported field rat meat consumption (Vera-Cabrera et al., 2015).

As previously mentioned, *M. leprae* in red squirrels in the British Isles is similar to that found in medieval human remains from England and Denmark (Avanzi et al., 2016). One hypothesis is that squirrels served as a zoonotic source for HD in medieval times (Inskip et al., 2017). There was substantial trade in squirrel fur, including between Scandinavia and the British Isles and further afield, with fur being highly prized for use in clothing (Veale, 2003), while squirrel meat was also valued. Only one paleopathological study to date has applied ancient DNA methods for *M. leprae* (RLEP primer) and *M. lepromatosis* (135bp *hemN* gene fragment primer). This was a study of the remains of four people from Ireland dated to the 10th–14th century and one person from the 15th–17th century, all of whom had osteoarchaeological signs of HD (Taylor et al., 2018). PCR was positive for *M. leprae* in three of the earlier remains and negative for *M. lepromatosis* in all five remains. Hansen’s disease declined in central Europe from the thirteenth century onward and was almost eliminated by the sixteenth century (Rawcliffe, 2006), leaving a zoonotic transmission hypothesis which is plausible but probably untestable either for *M. leprae* or *M. lepromatosis*.

## Conclusion

The recent discovery of *M. lepromatosis* and the attribution to this new species of cases of HD raised some initial doubts (Gillis et al., 2011; Scollard, 2016). While there does now appear to be consensus that *M. lepromatosis* is a second causal agent of HD (Sharma et al., 2020), a multitude of questions remain. Most of these can be answered using genomic methods to differentiate *M. leprae* and *M. lepromatosis* infection (Avanzi et al., 2020; Sharma et al., 2020). The most important immediate question in terms of public health, human disease burden, and HD prevention and control is the extent of *M. lepromatosis* infection in HD endemic countries and its contribution to HD incidence. In terms of individual persons affected by HD, although treatment outcomes for HD caused by *M. lepromatosis* appear similar to outcomes for the same forms of HD when caused (presumably) by *M. leprae*, the small number of *M. lepromatosis* HD cases described to date leaves a substantial gap in clinical knowledge. On these and the specific question of *M. lepromatosis* as a possible zoonotic source of HD, we propose the following steps:

1. A systematic review and data synthesis to determine clinical and other characteristics of all reported cases of HD attributed to *M. lepromatosis*.

2. PCR-based surveillance studies in current and new HD patients (regardless of the form of the disease), particularly in Mexico and Brazil but ideally in all endemic countries.

3. PCR-based surveys in Latin American countries to detect *M. lepromatosis* in newly captured animals or archived specimens from earlier studies, focusing on species, such as armadillos which are known to harbor *M. leprae* and which come into contact with humans. Experience with studies investigating *M. leprae* in wild armadillos in Brazil suggests that substantial variation in prevalence of infection might be expected (Deps et al., 2020); therefore, sufficiently large samples of animals from different geographic locations will be needed.

4. Whole genome sequencing of specimens from animal surveys and surveillance studies in Latin American countries to elucidate strains and determine the relatedness of *M. lepromatosis* (and *M. leprae*) in animals and humans (Avanzi et al., 2020).

Although improving our understanding of *M. lepromatosis* as a cause of clinical disease in humans should be our highest priority, the role of zoonotic transmission in HD caused by *M. leprae* proves the need to adopt a One Health perspective. This is particularly so in non-endemic countries, such as Mexico, where a large proportion of the small total number of new cases each year (<100 in 2020) could be attributable to zoonotic transmission. Conversely, knowing what proportion of the very large number of cases (~30,000 cases per year) in an endemic country, such as Brazil, might be caused by *M. lepromatosis* rather than *M. leprae*, as has been assumed to date, is of fundamental importance to public health.

## Author Contributions

PD conceived this article and the research question. SC reviewed the literature and wrote the first draft. PD and SC synthesized and interpreted data from the review, contributed to revisions, and approved the final version.

## Conflict of Interest

The authors declare that the research was conducted in the absence of any commercial or financial relationships that could be construed as a potential conflict of interest.

## Publisher’s Note

All claims expressed in this article are solely those of the authors and do not necessarily represent those of their affiliated organizations, or those of the publisher, the editors and the reviewers. Any product that may be evaluated in this article, or claim that may be made by its manufacturer, is not guaranteed or endorsed by the publisher.
